# Insecticidal roof barriers mounted on untreated bed nets can be as effective against *Anopheles gambiae* as regular insecticide-treated bed nets

**DOI:** 10.1038/s41598-023-48499-2

**Published:** 2023-12-12

**Authors:** Anthony J. Abbott, Agnes Matope, Jeff Jones, Vitaly Voloshin, Catherine E. Towers, David Towers, Philip J. McCall

**Affiliations:** 1 Vector Biology Department, Liverpool School of Tropical Medicine, Liverpool L3 5QA, UK; 2 Optical Engineering Group, School of Engineering, University of Warwick, Coventry, CV4 7AL UK

**Keywords:** Behavioural methods, Parasitic infection, Epidemiology

## Abstract

Barrier bednets (BBnets), regular bednets with a vertical insecticidal panel to target mosquitoes above the bednet roof, where they are most active, have the potential to improve existing Insecticidal Treated Bednets (ITNs), by reducing the quantity of insecticide required per net, reducing the toxic risks to those using the net, potentially increasing insecticide choice. We evaluated the performance of PermaNet 3.0 (P3) and untreated (Ut) bed nets with and without pyrethroid and piperonyl butoxide roof barriers in killing pyrethroid-resistant and susceptible *Anopheles gambiae,* simultaneously video-recording mosquito flight tracks. Bioassay results showed that treated roof barriers, particularly the longitudinal P3 barrier (P3L) could be an effective addition to a bed net: P3 + P3L were consistently significantly more effective than the reference P3 bednet while performance of untreated nets could be raised to equal that of the reference P3 following the addition of a P3 barrier. The BBnet’s potential to augment existing bednets and enhance their performance is considered.

## Introduction

With its most effective control tool, the insecticide-treated bednet (ITN), under serious threat from insecticide resistance, malaria control in Africa is at a critical stage. Evidence of ITNS losing efficacy against resistant vector populations has become widespread and malaria re-emergence is now a major threat^1–3^.

At the time of writing, there are 26 commercial ITN products listed with WHO prequalification, all of which use pyrethroids^4^. A pyrethroid is the only active ingredient (a.i.) on 16 of these nets, while on another seven, the added synergist piperonyl butoxide (PBO) improves pyrethroid efficacy by inhibiting the action of metabolic enzymes (cytochrome P450s) which can mediate pyrethroid resistance. Only three ITNs deploy a second insecticide from a different insecticide class: a pyrrole chlorfenapyr, or a juvenile hormone analogue, pyriproxyfen.

Clearly, PBO continues to extend the effective lifespan of pyrethroids for use as net treatments^5^ and the absence of cross resistance between any major insecticide class and chlorfenapyr secures the immediate future for ITNs. Historically, however, the arrival of resistance has been inevitable following the large-scale implementation of new net treatments^6^, especially when genetically diverse and interconnected populations are being targeted^.^ If resistance to chlorfenapyr were to emerge today^7^, bednets would be left dependent on an insecticide class against which the target populations are already highly resistant^8^, with poor prospects for expanding the range of potential treatments.

Expansion of the list of permissible net treatments is essential if we are to ensure a future for ITNs in malaria vector control. However, the range of possible treatments remains constrained by the need to minimize risks to the sleeper, while the higher cost of new insecticides could make some treatments economically unviable. There are no alternative vector control options that can deliver levels of protection comparable to ITNs, both for those in the community that use nets routinely and those without nets. Failure to ensure their future would be disastrous for Africa.

**Figure 1:**
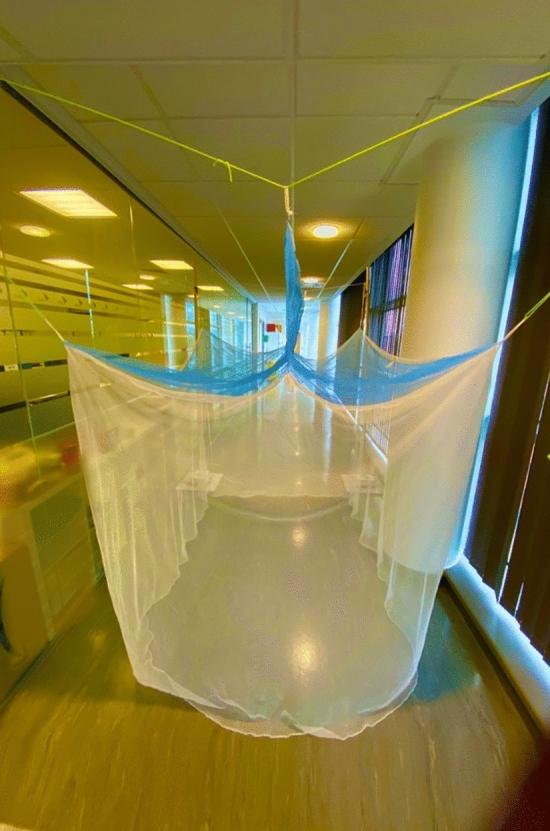
Example of a longitudinal Permanet 3.0 polyethylene barrier bednet. The blue netting on the barrier and roof is 100 denier polyethylene net with deltamethrin incorporated at 120 mg/m^2^ and PBO at 750 mg/m^2^. The white netting on the sides is 75 denier polyethylene with deltamethrin incorporated at 84 mg/m^2^ (Photo by PJ McCall).

In response to this challenge, Barrier Bednets (BBnets), bed nets with barriers or panels of insecticide treated netting on the ‘roof’ were designed with the hope of improving the performance of existing bednets against pyrethroid-resistant mosquitoes and to permit the safe deployment of active ingredients^9^. Upright roof barriers intercept mosquitoes above the bednet roof, where *An. gambiae s.l.* are most active^10^ and where the insecticide-treatment cannot contact the sleeper (Fig. 1). The possibility that deploying a more effective insecticide on the barrier could result in a net capable of performances that are equal to, or even better than, those of a standard ITN is an attractive prospect. Such a BBNet could potentially increase the range of candidate insecticides, by making newer expensive insecticides affordable, allowing higher concentrations of the a.i. or permitting the use of some insecticides that would be considered unsafe or unsuitable for direct skin contact if the sides and roof of the bed net were treated. In addition to increasing treatment choice, BBnets would require less insecticide per net, which translates as safer cheaper nets, less toxic waste, and fewer impacts on non-target fauna.

Following the initial demonstration of barrier bednet efficacy against a wild pyrethroid resistant population in Burkina Faso^9^ we report here on laboratory tests investigating the potential of different roof barrier designs of PBO-treated netting. We used the same infra-red video tracking system as earlier studies^9,10^ to video-record the interactions between *An. gambiae s.l.* from a laboratory colony as they flew freely in a large climate-controlled room, responding to a human volunteer host within different BBnet variants. This allowed considerable control over the experimental setup without interfering with the spatial behavior of the mosquitoes as they interacted with the ITN while responding to the host.

## Results

The study started in late 2019 in UK and was forced to stop as the first Covid lockdown measures were implemented in March 2020, necessitating a reassessment of experimental plans to ensure we could complete enough bioassays to compare the range of BBnet variants. Consequently, the resistant Tiassalé strain was tested in six repeat assays with all BBnet variants, but the susceptible Kisumu strain was tested only with transverse BBNet types, in six repeat assays for all variants except P3 + P3T, for which only 4 repeat assays were completed.

Following completion of the assays, susceptibility to deltamethrin and permethrin of the colonies of both strains were tested in May 2020 by LITE using standard WHO tube tests to determine levels of pyrethroid susceptibility: Kisumu was fully susceptible to both, with 100% mortality recorded in all tests. The Tiassale strain recorded mortality rates post exposure of 13% and 8% to deltamethrin and permethrin, respectively, and the colony underwent re-selection, beginning in May 2000 from which time it was no longer available.

**Figure 2:**
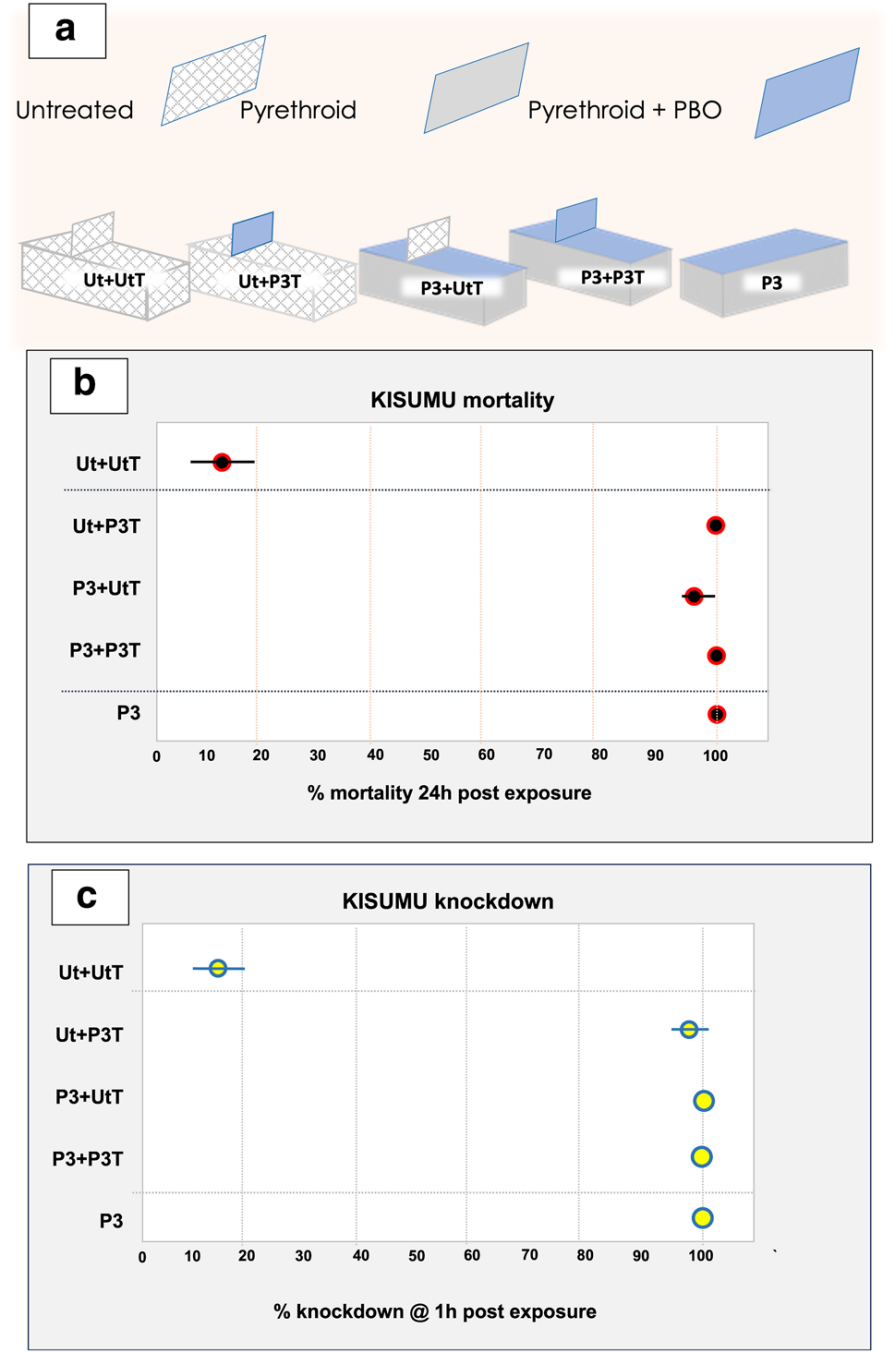
Mortality and knockdown rates of pyrethroid susceptible *Anopheles gambiae* Kisumu strain at transverse barrier bednet variants. (**a**) schematic illustrating the composition of each of the BBnets tested; (**b**) Mortality and (**c**) knockdown rates following the room-scale tests, as determined from videos recorded during tests and final counts at the termination of the 120 min assay (mean and CI; n = 6 repeat tests /treatment, except P3 + P3T (n = 4)). All images were created by the authors.

**Figure 3:**
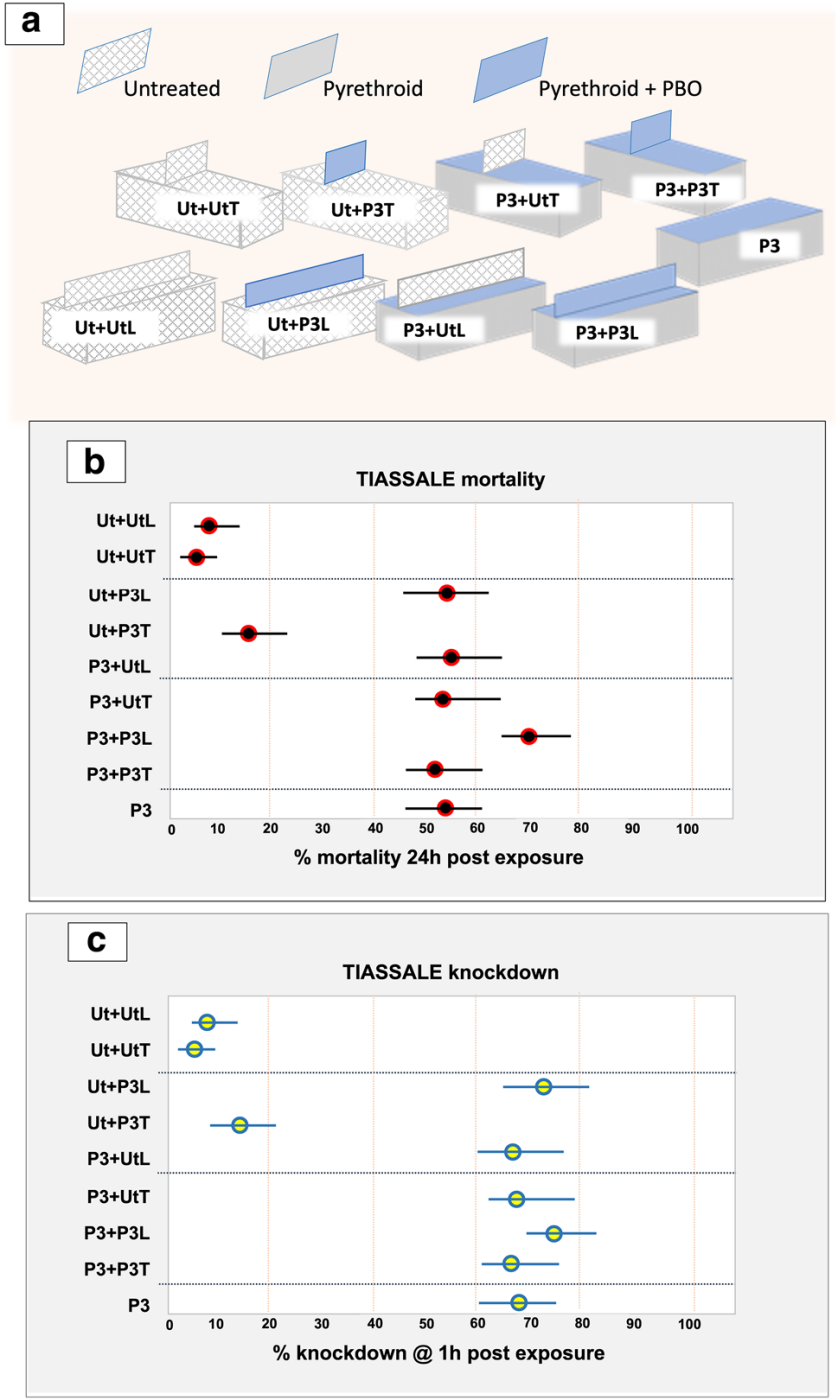
Mortality and knockdown rates of pyrethroid resistant *Anopheles gambiae* Tiassale strain at transverse and longitudinal barrier bednet variants. (**a**) schematic of the composition of the BBnet variants tested. 1. (**b**) Mortality and (**c**) knockdown rates following the room-scale tests, as observed in videos recorded during the 120 min tests and final counts at the termination of the 120 min assay (mean and CI; n = 6 repeat tests /treatment). All images were created by the authors.

BBnet variant names are based on their composition, as illustrated in Figs. 2a and 3a: Bednet treatment (Untreated or Permanet 3) + barrier treatment (Ut or P3) and shape (transverse ‘T’ or longitudinal ‘L’); *e.g.,* Ut + P3L is an untreated bednet with a longitudinal barrier of Permanet 3.0 (P3) netting.

### Performance of Transverse P3-BBnets against pyrethroid susceptible mosquitoes

The mortality and knockdown rates of tests with pyrethroid susceptible Kisumu strain mosquitoes at nets with transverse barriers are summarized in Fig. 2. The unmodified P3 net killed 100% of mosquitoes in all but one test repeat, where it killed 97%. All barrier bednet combinations with at least one P3-treated surface knocked down 91–100% of susceptible mosquitoes within 1 h and killed 92–100% within 24 h (Fig. 2b, c). Comparing the knockdown and mortality rates of the three BBnets with at least one P3 section, all the exposed Kisumu mosquitoes died within 24 h post-exposure except for a few in the Ut + P3T. Although not statistically significant, the performance of the untreated bednet with a transverse P3 barrier (Ut + P3T) was comparable to a standard treated Permanet 3.0 ITN, indicating that the barrier’s position, above the net and directly over the human sleeper’s torso, is well placed to target *Anopheles sp* mosquitoes as they attempt to reach the human inside.

### Performance of the BBnet variants against pyrethroid resistant mosquitoes

The mortality and knockdown rates of tests with Tiassale strain mosquitoes at transverse and longitudinal BBnets are summarized in Fig. 3.

Five BBnet variants achieved mortality rates greater than 50%, levels similar to the unaltered basic P3 reference (Fig. 3b, c). The lowest performance was Ut + P3T, an untreated bednet with a shorter transverse P3 barrier. Tiassale mosquitoes were twice as likely to die when exposed to P3 + P3L and 63% less likely to die when exposed to Ut + P3T compared to P3 (P3 + P3L: OR = 2.09; 95% CI: 1.21, 3.64; *p* = 0.0086, and Ut + P3T: OR = 0.37; 95% CI: 0.20, 0.67; *p* = 0.0013).

Four of the five variants with efficacy comparable to the P3 (knockdown > 70%; mortality > 50`%) comprised a barrier mounted on a complete P3 base. The fifth variant, Ut + P3L, was an exception, as it achieved mortality rates comparable to the other four variants and the P3 reference although its bed net base was untreated. The P3L barrier on a P3 bed net base performed very well, exceeding mortality rates of the P3 in all assay repeats, a difference that was significant despite wide confidence intervals (OR 2.09; 95% CI 1.21, 3.64;* p* = 0.0086). No significant differences were found between the other nets and the P3 net.

### Contact rate and duration of contact

**Figure 4:**
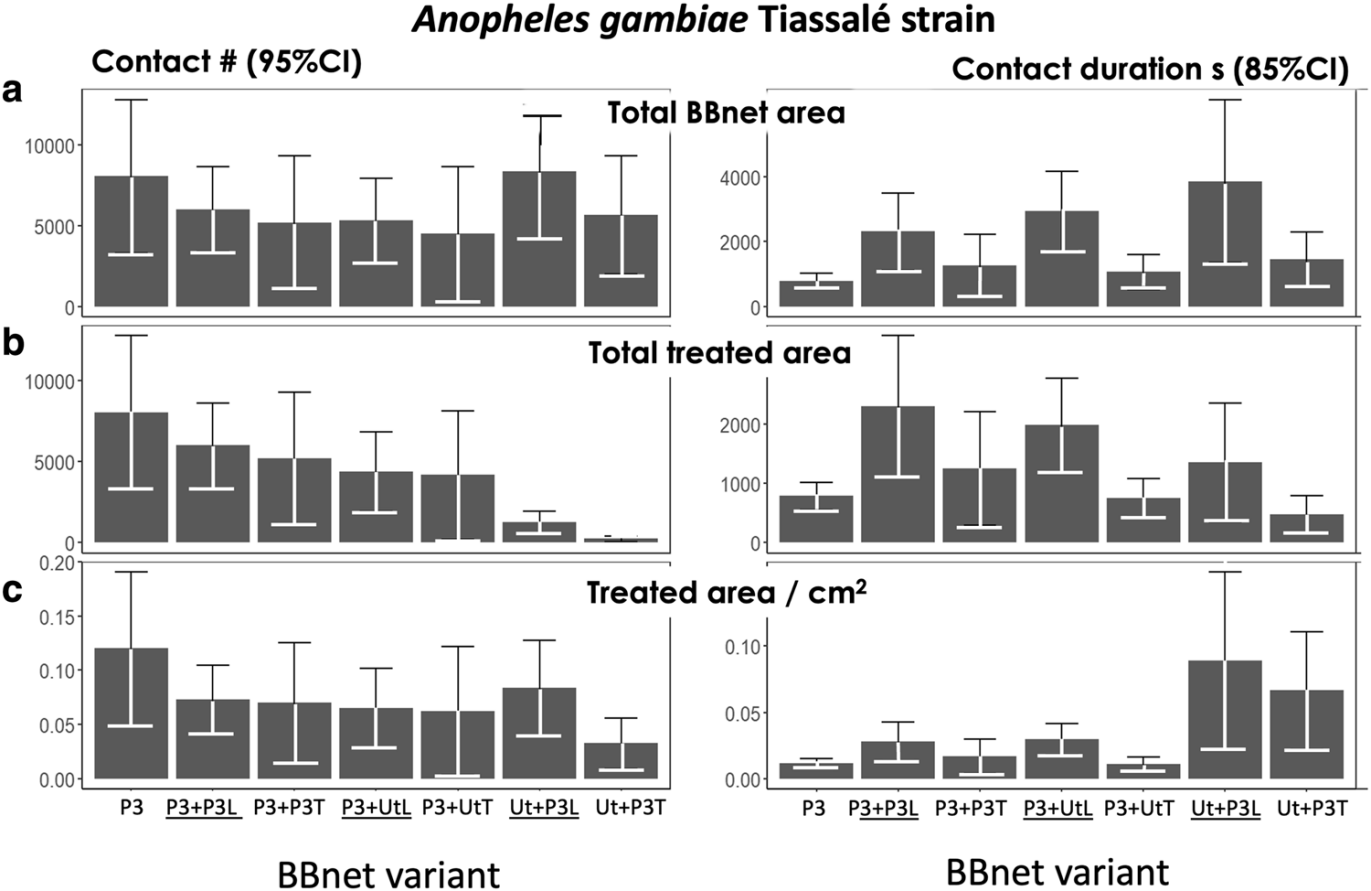
Mean total numbers and durations of contact by *Anopheles gambiae* Tiassalé strain with netting during the 120 min assay for (**a**) the entire surface area of netting (treated and untreated) on the BBnet; (**b**) the entire treated net surface area only (**c**) per cm^2^ of the entire area of treated netting.

The mean total number of contacts (and CI) made and the duration of that contact for each BBnet variant are given for Tiassale strain mosquitoes in Fig. 4, which shows the total number and duration of contacts on any part of the BBnet (Fig. 4a), on the treated sections only (Fig. 4b) and per cm^2^ of treated net (Fig. 4c). See also data and results, summarised in Tables S1 and S2). The duration of contact with the total net surface (all untreated and treated net surfaces, including barrier, Fig. 4a) during exposure was longer (at least 1465.66 s) in all net variants compared to P3. However, these differences were significant only with P3 + P3L, P3 + UtL and Ut + P3L).

The duration of contact with the treated net surfaces (Fig. 4b) was significantly longer (for Ut + P3L, P3 + UtL, and P3 + P3L compared to P3 net alone (Table S1, Ut + P3L: mean difference = 1465.99; 95% CI: 533.44, 2398.54; p = 0.0031, P3 + UtL: mean difference = 1691.48; 95% CI: 885.42, 2497.54; p < 0.0010, and P3 + P3L: mean difference = 1794.62; 95% CI: 1028.98, 2560.26; p < 0.0010). Although we still do not know the precise minimum contact time required for a polyester ITN to deliver a lethal dose of an insecticide, increased contact duration is associated with higher kill rates^10^. Summary statistics ae shown in Table S1.

## Discussion

The results of these tests, although not fully conclusive, demonstrate the potential of simple roof mounted bednet barriers for malaria vector control. The bioassays clearly demonstrate that treated longitudinal barriers are likely to greatly improve the performance of the bednet beneath them. This ability could extend as far as ‘converting’ an untreated net into an effective ITN or ‘restoring’ an intact aged net simply by the addition of a treated barrier.

The key to the barriers’ ability to kill so efficiently is almost certainly their position on the roof: Here they are located directly above the supine human inside the bednet, where activity by incoming mosquitoes is greatest^10^ maximizing the chance of encountering and interrupting flight paths of the majority of *An. gambiae* visiting the net. This is illustrated by a comparison of the levels of contact at a longitudinal barrier with a total area of insecticidal netting of 7600 cm^2^ and a Permanet 3.0 bednet which has 8 times greater area of treated net, 62,200 cm^2^. A comparison of the total duration of contact at the treated sections on a Ut + P3L BBnet and the reference P3 bednet (Fig. 4b), shows that despite the disparity in insecticidal surface area the longitudinal P3 barrier accumulated 1374 s duration from 1273 contacts, greatly exceeding the 792 s duration, from 8049 contacts, accumulated by all the treated net surface area at the Permanet 3.0. This was equivalent to contacts rates at the L barrier with a total duration of 0.9 s/cm^2^ compared with the P3 ITN which was 0.11 s/cm^2^ (Fig. 4c). The duration rates are remarkably similar, despite the difference in area.

The ITN has 8 times greater surface area loaded with insecticide, most of which does not contribute to its impact (Fig. 4). Moreover, mosquitoes orienting towards the bednet roof probably arrive first at the barrier/roof and may already have picked up a lethal dose of insecticide before they visit any other part of the net. This begs the question of whether an effective barrier on an untreated bednet base could kill mosquitoes as well as a regular treated bednet or put another way, could an untreated net reproduce the killing effect of a standard ITN simply by the addition of a treated barrier? These results certainly suggest it is possible, with an appropriate insecticide treatment. As Fig. 4a shows, the duration of contact with the total net surface (all untreated and treated net surfaces, including barrier) during exposure was longer in all net variants compared to P3, but the differences were significant only with P3 + P3L, P3 + UtL, Ut + P3L. The killing performance of the untreated bednet with a long P3 barrier (Ut + P3L) was comparable to a standard treated Permanet 3 ITN (Fig. 3).

To date, the BBnet has been tested mainly with insecticides that would be permissible on a standard bednet, except for the use of fenitrothion in Burkina Faso study, when the BBnet (insecticidal barrier with untreated bed net base) performed very well, increasing lethality without compromising personal protection^9^.

The pattern of movement around a bednet where most mosquito activity occurs on or above the roof, is a response to olfactory and thermal cues rising from the host below, and Sutcliffe and Yin^11^ reported that the pattern disappeared when a breeze was blown across the host, dissipating any rising attractants, and eliminating the focus of activity on the net roof. This is true and it is widely known that sleeping with a steady low continuous flow of air from a reliable electric fan prevents mosquitoes from landing to the extent that it is possible to sleep comfortably without any bednet. However, at present few communities in endemic malaria zones are likely to have access to affordable power needed to run an electric fan all night every night, while security concerns are likely to continue to overrule any desire for comfort when deciding to open a window. Hence, until such basic improvements are widespread, this is unlikely to be a factor affecting the performance of BBnets.

The poor performance of the transverse barriers against resistant mosquitoes was unexpected. As already mentioned, in our earlier field study, we found that a transverse barrier treated with the organophosphate fenitrothion was highly effective against a wild population of pyrethroid-resistant *An. gambiae s.l.*, in Burkina Faso increasing killing approximately 34% more than a standard bednet^9^. Here, it performed well against susceptible mosquitoes (Fig. 2) but did not improve the performance of bednets against resistant mosquitoes compared to the negative controls, despite the high duration of contact at the treated barrier in the Ut + P3T Fig. 4c). Differences in the insecticides used could explain this at least in part. In the field study where the transverse barriers were effective, they carried a highly effective insecticide, either deltamethrin against susceptible mosquitoes, or an organophosphate, fenitrothion against the wild highly resistant vector population in Burkina Faso^9^. In both cases, the insecticide’s effect was rapid following brief contact with a treated net. In studies where the transverse barriers performed poorly, as with Tiassale in the present study, impact depended on the synergist piperonyl butoxide (PBO) disabling the resistant mosquito’s p450 resistance mechanism before sufficient insecticide could impact. Although the minimum contact times needed to pick up a lethal dose for each of these treatments are not known, it is conceivable that the faster acting insecticides were more suited to delivery on a barrier, as the threshold for a lethal level of contact is brief (< 50 s)^10^. The loss of the highly resistant Tiassale colony when it was too late to repeat the experiments of was most unfortunate, given the importance of demonstrating an impact with resistant mosquitoes. However, as mentioned above, the Burkina Faso study where fenitrothion-loaded T-barriers were effective against highly resistant wild/natural vector population serves to demonstrate that with an appropriate insecticide, BB-nets can be effective against highly resistant vectors^9^.

An estimated 79% of malaria cases are transmitted when people are in bed^12^, making the value and importance of ITNs in malaria prevention very clear. With careful management, ITNs can remain the primary means of reducing indoor malaria transmission in Africa for many more years. Standard bednet shapes can safely deliver only a very limited range of insecticides, seriously limiting options for management of insecticide resistance. Simplicity has been integral to ITN’s success, but nets do not need much sophistication to improve them. They employ the sleeper’s attractants to lure potentially infectious mosquitoes to the net surface where they are rapidly killed on contact while the sleeper is protected from bites behind a protective insecticidal screen^10^. Requiring only minimal change to the basic design, a roof barrier exploits this further. Of note here is the improvement in performance that a treated barrier contributes to the base bed net’s efficacy. This is apparent in Fig. 3b where the long P3 barrier raised the untreated bed net’s mortality rate from minimal to a rate equivalent to that of a standard P3, and the standard P3 from a mean mortality rate of 54% to 70%. If insecticide were to be delivered only on the roof and barrier it would reduce the risk of the occupant’s skin becoming irritated by insecticide picked up during entry and exit from the protective net, further enhancing its advantages.

**Figure 5:**
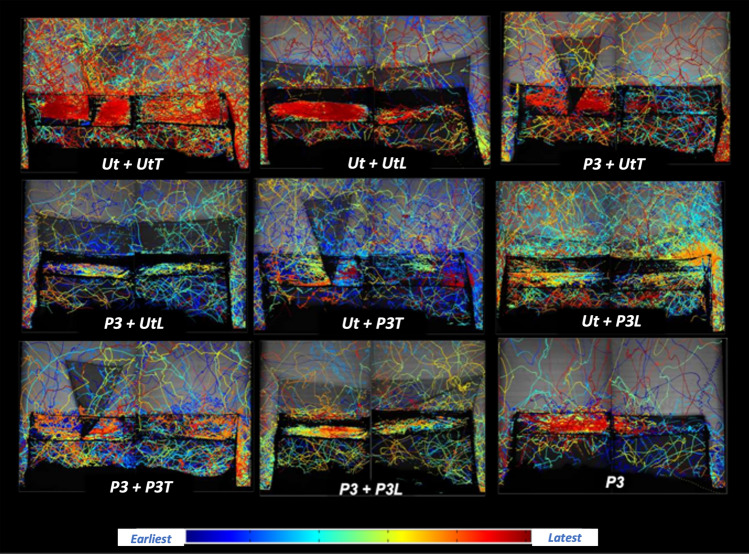
Examples of 120-min composite images showing all flight tracks of *An. gambiae* Tiassale strain at each of the BBnet variants. In all images, the sleeper is lying on their back, with their head at the left. Each colored track is the path of a single mosquito flight event, (25 mosquitoes released simultaneously) in all tests and color-coded according to time of appearance as shown in the key: blue tracks at the start through to red at the end of the 120-min test.

Given the importance of the net roof, and the region above it to the lethality of any ITN, it would make sense if attempts to improve ITN killing efficiency were to focus on this region. As Fig. 5 shows, none of the BBnets altered the preference for the bed net roof. The low levels of mosquito activity at the vertical sides and ends of the bednet^10^ suggest that the best we can do at these net locations, where most physical damage occurs, is to improve the quality of the material used in manufacturing the net to make stronger more durable nets. Interest in improving nets has increased such that most of those involved in iTN deployment and development recognize the need to make nets that are more durable not only in use but also when in storage prior to use^13–16^, nets that produce fewer toxic residues when discarded and are generally more recyclable^17^. Some have prioritized durability over insecticide delivery^18^. This is the first study to demonstrate how an insecticidal barrier can improve ITN performance, and how it can increase the options for different ITN designs. Moreover, we have shown that this can be achieved without the use of an additional active ingredient. The BBnet uses our knowledge of the habits of *Anopheles* around an occupied bed net by placing treated netting precisely where the mosquitoes are most likely to occur when they first arrive at the net. Existing bed nets do not go far enough to achieve this.

Better bednets should mean a lot more than simply bednets with active ingredients that are effective against the target vector population, as this is surely the least any bednet should be. It is often a shock when the delicate fibers comprising the flimsy mesh that an ITN is made from are first seen. Sufficient knowledge of the entomological mode of action of ITNs already exists to enable us to identify the location of regions of the net where a number of important vector mosquito species are most likely to arrive^19–21^ and where the insecticide is best positioned to maximise contact with mosquitoes^11^, the areas most vulnerable to physical damage that would benefit from tougher net fibre, and areas that are rarely visited by mosquitoes. Recent research suggests that most bed nets, including the new bi-treated nets, have similar entomological modes of action^22^. Can this range of knowledge not be used to design better bed nets, durable ITNs that are optimised to kill mosquitoes and prevent malaria transmission in rural Africa, rather than simply the cheapest designed products that tick the boxes required to complete a WHO form?

## Materials and methods

### Mosquitoes and bioassays

Mosquitoes used in all tests were obtained from LITE All colonies were maintained, and bioassays were performed in a climate-controlled unit at LSTM (27 ± 2 °C, relative humidity 70 ± 10%) measuring 5.6 m × 3.6 m in area and 2.3 m high, using 2–7-day old unfed adult females from two *An. gambiae* s.l colonized strains. The Kisumu strain originated in Kenya in 1975 and is susceptible to pyrethroids and all other insecticides. The Tiassalé strain was established at LSTM in 2013, from material collected in Côte d’Ivoire. It is pyrethroid resistant and resistant to DDT and carbamates, conferred by a combination of target site mutations and P450 enzymes^23^. The colonies are maintained under a standardised rearing regime and Tiassalé strain undergoes continual selection with deltamethrin by Liverpool Insect Testing Establishment (LITE). Mosquitoes were obtained from this central facility as required (https://lite.lstmed.ac.uk/mosquito-colonies).

Mosquitoes used in bioassays were deprived of sugar and water for 24 h and 4 h respectively before bioassays and transferred to a 300 ml paper cup to the bioassay room to acclimatize for 1 h prior to the start. Tests began 1–3 h after the start of the scotophase with the release of 25 test mosquitoes into the room (5.6 m × 3.6 m in area, 2.3 m high) and ran for 2 h, after which time the room was searched thoroughly to count and collect live, dead, or knocked down mosquitoes. All live mosquitoes were aspirated into a cup and provided with 10% sugar water in a separate room where mortality was recorded at 1 h and 24 h after the end of the experiment.

We used 27 volunteers (11 male, 16 female) aged between 22 and 61 years, all recruited from within LSTM and representing a range of ethnicities. Each person volunteered twice or more, acting as host for a different net on the second or subsequent occasion. The bioassay room was cleaned on Friday and an untreated net was tested on the next day (Monday) to ensure the room was clean. If the mortality was unexpectedly high, the bioassay room would be cleaned again.

Volunteers lay without shoes but clothed in their own trousers and t-shirt and uncovered within the bednet. They were requested to eschew strongly aromatic or spiced foods and scented personal hygiene products for 24 h prior to each experiment. Since the tests were also video recorded for tracking, volunteers were requested to remain as still as possible for the duration of the experiment.

### BBnet variants

In all tests, rectangular bednets measuring 1.9 m × 0.8 m ×  ~ 1.0 m tall were used as the standard bednet. Treated nets were Permanet 3.0 (Side walls of 75 denier polyester, deltamethrin 2.1 g/kg ± 25%; roof of 100 denier polyethylene, deltamethrin 4.0 g/kg ± 25% and PBO 25 g/kg ± 25%; Vestergaard, Lausanne). Barriers comprised sections cut to size from the roof of a Permanet 3.0. Untreated polyester nets were used as untreated controls, and matched Permanet 3.0 nets as closely as possible fiber thickness and hole size. Before being used in experiments, untreated nets were first tested by WHO cone test to confirm the absence of any insecticidal effect, and all nets were hung for 4 weeks before use to allow evaporation of any potentially repellent or attractant volatile odors.

Two styles of barrier were investigated, a transverse (from elbow to elbow of the supine sleeper beneath) and a longitudinal barrier (from head to toe; see Fig. 1), designs derived from earlier bioassay in the field or from mathematical models comparing their potential^9^. Transverse barriers were positioned off-center on the roof above the sleeper’s stomach or torso at the 30:70 division of the bednet’s length, where mosquito activity is known to be greatest^10^.

To facilitate image capture on the top of the bednet, the net roof was tilted on its long axis when facing the cameras to ensure all mosquito activity on the roof was visible^9,10^. Hence, the height of the roof above the mattress was 0.80 m at the front and 1 m at the rear (when viewed/recorded from the front). To ensure the top edge was horizontal when mounted on this roof, the transverse barrier was 40 cm tall at the rear and 60 cm at the front of the net. The longitudinal barrier was 0.4 m higher than the roof and ran the length of the bednet in the center of the roof.

The basic structure of transverse and longitudinal barriers was similar in all tests as illustrated in Figs. 2a and 3a. BBnets were coded according to the identity of the treatment on the bed net followed by the treatment on the barrier, and the style of barrier (Fig. 3a) as follows:

- Untreated net + untreated long barrier **Ut + UtL**
- Untreated net + untreated transverse barrier**UT + UtT**
- Untreated net + long P3 barrier**Ut + P3L**
- Untreated net + transverse P3 barrier**Ut + P3T**
- Permanet 3.0 NET + untreated barrier**P3 + UtL**
- Permanet 3.0 NET + untreated P3 barrier**P3 + UtT**
- Permanet 3.0 NET + long P3 barrier**P3 + P3L**
- Permanet 3.0 NET + transverse P3 barrier**P3 + P3T**
- Permanet 3.0 with no barrier (standard P3 net)** P3**

Twenty-five non-bloodfed female mosquitoes were used in each bioassay. Longitudinal BBnet designs were tested in 6 repeats with both susceptible and resistant strains, but the transverse barriers were tested in 6 repeat tests with the Kisumu susceptible strain only, with the exception of P3 + the result of time limits as the first COVID-19 lockdown approached.

### Tracking protocols

All bioassays were recorded for subsequent analysis of bednet contact using the video tracking system installed in Liverpool as described previously in detail^24^. The tracking system comprised two identical cameras (Ximea CB120RG-CM with Canon EF 14 mm f/2.8L II USM lenses) with a custom LED ring attached (12 × OSRAMTM SFH 4235 infrared LEDs (peak wavelength 850 nm)). These were aligned with a Fresnel lenses per camera mounted at 1.2 m distance from the camera lens, and a retroreflective screen (plywood board, with 3M Scotchlite High Gain 7610 tape) mounted behind the bednet, 2 m from the Fresnel lenses. Recordings were made at 50fps using StreamPix software (www.norpix.com) and files saved in a .seq file format.

Recordings were segmented, creating a text file containing the coordinates and identity of each track, and additional noise recorded (such as movement from the volunteer) was cleaned up using the bespoke ‘seqfile processing’ software. The cleaned position files were then analyzed using bespoke MatLab (mathworks) software which defined the net regions. Continuous tracking of individual mosquitoes was not possible since the whole room is not in view, so each flight track was analyzed from the point of entry until leaving the field of view. The area recorded was separated into different regions breaking up different parts of the bednet and the surrounding area, with the number and duration of net contacts (bednet treated/ untreated or barrier treated/ untreated) recorded for each.

### Statistical analyses

Descriptive statistics were generated using the number of observations, mean, and standard deviation (SD) for continuous variables, the number and percentage of observations for categorical variables.

The duration of contact and the number of contacts made with each net were analysed using Linear Mixed Models (LMM). Mixed models were employed to account for the correlation between measurements from the same sleeper. Net type (nine categories: P3, P3 + P3T, P3 + P3L, P3 + UtT, P3 + UtL, Ut + P3T, Ut + P3L, Ut + UtT, Ut + UtL), maximum number of mosquitoes in contact with the net, the position of the volunteer’s head (two categories: left and right) and the possible significant interactions between these factors were included as fixed effects, and volunteer as a random effect.

For 1 h KD and 24 h mortality, a logistic regression model with a logit link function was fitted. The models for the Kisumu and Tiassale strains were fitted separately. For the Kisumu strain, the penalised likelihood estimation was employed to account for the complete separation that was observed for some net groups in the study^25^. The fixed effects considered included net type, duration of contact with the net/treated surface (Tiassale strain model only), and their interaction.

Statistical analyses were performed using R Version 4.2.1 and RStudio^26,27^. All the models were fitted using the “lme4” package while net contrasts were explored using the “emmeans” package^28,29^. Model selection was implemented on the full models described above to obtain the most parsimonious model. Likelihood ratio tests under the restricted maximum likelihood estimation were employed to determine the statistical significance of the random effects. The fixed effects for the ‘best’ fit models were selected based on model fitness determined using the Akaike information criterion and the residuals based on the “DHARMa” package^30^. Data were plotted using packages “ggplot2” and “ggfortify”^31,32^.

All the net types were compared to P3 (as reference), and the Dunnett multiple test adjustment procedure was employed to control for the probability of making false positive findings. The mean differences and odds ratios (OR) together with their corresponding 95% confidence interval (Cl) were generated for the net comparisons from the LMMs and logistic regression models, respectively. All the statistical tests were conducted at 5% Significance level.

### Ethical considerations

All of the experimental protocols used in this study are approved by the Research Ethics Committee of the Liverpool School of Tropical Medicine. All volunteers provided written informed consent before participation and all methods were carried out in accordance with relevant guidelines and regulations.

### Supplementary Information

Supplementary Information.

## Data Availability

All data are provided in the supplementary information.
