# Supplementary material for: Insecticidal roof barriers mounted on untreated bed nets can be as effective against Anopheles gambiae as regular insecticide-treated bed nets
Source: Sci Rep. 2023 Dec 12;13:22080. doi: 10.1038/s41598-023-48499-2 (PMC10716170; doi:10.1038/s41598-023-48499-2)
Supplement: Supplementary file 1 — Supplementary Information. [file 41598_2023_48499_MOESM1_ESM.docx]

**SUPPLEMENTARY INFORMATION**

**Abbott *et al* Barrier Bednets for malaria vector control**

**Fig S1. Tracked regions where losses of multiple flight tracks required adjustment**

The tracking system used here was the later refined system using a retro-reflective screen (RRS) to give more uniform and diffuse illumination (‘V2’; Voloshin *et* al, 2020). The original approach was back-lit with the light making a single pass through the measurement volume (‘A1’; Angarita-Jaimes *et al.*, 2016). Using the RRS setup here with a BBNet we found that in certain sections, the position of a barrier added another layer of netting such that the line of sight of the camera passed through three layers of netting – and due to the retro-reflective imaging setup – these layers are double passed before the image is obtained at the camera. Such image areas gave few or no detected tracks (A and C in Fig S1). Each layer of net can be thought of as attenuating the optical signal and hence using RRS imaging with 3 net layers gives 6 traversals of net between the light source and the camera. For more conventional bednet setups when imaging through 2 layers of net (4 traversals), the RRS setup gave improved performance compared to back-lit imaging due to the diffuse nature and more uniform illumination produced. These benefits are lost when an additional bednet layer (the barrier) is added as the optical signal is reduced to give insufficient image contrast to segment the mosquito shadows from the already low optical signal. The back-lit imaging setup may have given better results for the BBNet case under study here, however, back-lit imaging requires 2 large area Fresnel lenses per camera and the lenses were already in use for studies in west Africa and committed to remain there for a lengthy period.


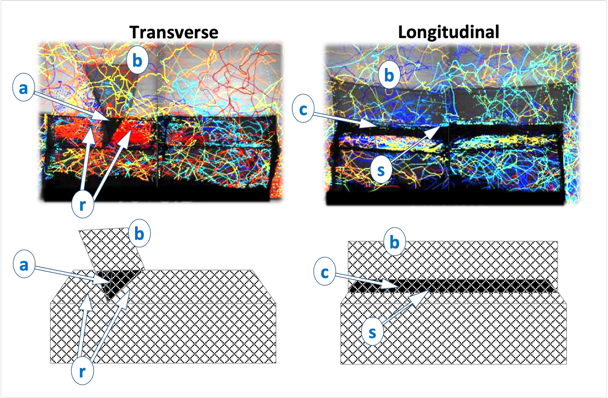
To compensate for tracks lost during recording, track counts were adjusted as follow**s:**

Transverse barrier: as mosquito activity in Section A was viewed through three layers of netting, much activity was concealed, and counts affected in areas equivalent to a mean of 10.5% of the area in the head end section of the roof (R) and 14.2% of the barrier (B). The final net contact counts for the barrier and the roof-head end were increased proportionately. Longitudinal barrier: here, Section C was obscured by 3 net layers in areas equivalent to mean rates of 42% of the roof (R), 29% the barrier (B) and the upper 50% of the sides: net contact counts were increased proportionately. Accuracy in adjustment was aided by the knowledge that distribution of tracks on bednets is equal on left and right sides of the host (Parker et al, 2015).

| **Net** | **Strain** | **n** | **1h KD** | | | | **24h Mortality** | | | |
| --- | --- | --- | --- | --- | --- | --- | --- | --- | --- | --- |
|  |  |  | **Number KD** | **% KD** | **95% Lower CI** | **95% Upper CI** | **Number dead** | **% dead** | **95% Lower CI** | **95% Upper CI** |
| P3 | Kisumu | 146 | 146 | 100.00 | 100.00 | 100.00 | 146 | 100.00 | 100.00 | 100.00 |
| P3 | Tiassale | 147 | 100 | 68.03 | 59.91 | 76.15 | 80 | 54.42 | 46.30 | 62.54 |
| P3 + P3L | Tiassale | 147 | 112 | 76.19 | 68.83 | 83.55 | 105 | 71.43 | 64.06 | 78.79 |
| P3 + P3T | Kisumu | 98 | 98 | 100.00 | 100.00 | 100.00 | 98 | 100.00 | 100.00 | 100.00 |
| P3 + P3T | Tiassale | 143 | 95 | 66.43 | 58.20 | 74.66 | 78 | 54.55 | 46.31 | 62.78 |
| P3 + UtL | Tiassale | 139 | 94 | 67.63 | 59.30 | 75.95 | 78 | 56.12 | 47.79 | 64.44 |
| P3 + UtT | Kisumu | 137 | 137 | 100.00 | 100.00 | 100.00 | 137 | 100.00 | 100.00 | 100.00 |
| P3 + UtT | Tiassale | 137 | 90 | 65.69 | 57.28 | 74.10 | 75 | 54.74 | 46.33 | 63.15 |
| Ut + P3L | Tiassale | 139 | 105 | 75.54 | 67.18 | 83.90 | 75 | 53.96 | 45.60 | 62.32 |
| Ut + P3T | Kisumu | 143 | 140 | 97.90 | 95.18 | 100.63 | 139 | 97.20 | 94.48 | 99.93 |
| Ut + P3T | Tiassale | 144 | 65 | 45.14 | 39.00 | 51.28 | 24 | 16.67 | 10.53 | 22.81 |
| Ut + UtL | Tiassale | 148 | 8 | 5.41 | 0.97 | 9.84 | 12 | 8.11 | 3.67 | 12.54 |
| Ut + UtT | Kisumu | 142 | 16 | 11.27 | 5.50 | 17.04 | 20 | 14.08 | 8.31 | 19.86 |
| Ut + UtT | Tiassale | 142 | 5 | 3.52 | 0.00 | 7.56 | 9 | 6.34 | 2.30 | 10.38 |

**Table S1. Descriptive statistics for knockdown and mortality by strain and net type** ( KD = knockdown, n = sample size per group and CI = confidence interval.

| **Term** | **Odds Ratio [95% Confidence Interval], p-value** | | | | | |
| --- | --- | --- | --- | --- | --- | --- |
|  | **1h KD** | | **24h mortality with all groups** | | **Treated surfaces groups only (Tiassale)** | |
|  | **Kisumu** | **Tiassale** | **Kisumu** | **Tiassale** | **1h KD** | **24h mortality** |
| (Intercept) | 293.00  [42.98, 36877.75], <0.0010* | 2.13  [1.51, 3.03], <0.0010* | 293.00  [42.98, 36877.75], <0.0010* | 0.11  [0.05, 0.26], <0.0010* | 1.79  [1.22, 2.66], 0.0034* | 0.11  [0.05, 0.27], <0.0010* |
| Net: P3 + P3T ^a^ | 0.67  [0.00, 124.48], 0.8435 | 0.88  [0.53, 1.48], 0.6339 | 0.67  [0.00, 124.48], 0.8435 | 1.07  [0.64, 1.77], 0.8003 | 0.80  [0.48, 1.36], 0.4087 | 1.08  [0.65, 1.79], 0.7751 |
| Net: P3 + P3L ^a^ |  | 1.50  [0.90, 2.53], 0.1197 |  | 2.09  [1.21, 3.64], 0.0086* | 1.09  [0.60, 2.01], 0.7776 | 2.18  [1.22, 3.92], 0.0087* |
| Net: P3 + UtT ^a^ | 0.94  [0.01, 173.65], 0.9748 | 0.81  [0.48, 1.35], 0.4108 | 0.94  [0.01, 173.65], 0.9748 | 1.15  [0.70, 1.92], 0.5785 | 0.81  [0.48, 1.36], 0.4246 | 1.15  [0.69, 1.92], 0.5853 |
| Net: P3 + UtL ^a^ |  | 0.98  [0.60, 1.61], 0.9421 |  | 1.53  [0.91, 2.58], 0.1085 | 0.75  [0.43, 1.33], 0.3270 | 1.57  [0.91, 2.72], 0.1027 |
| Net: Ut + P3T ^a^ | 0.14  [0.00, 1.43], 0.1052 | 0.39  [0.24, 0.62], <0.0010* | 0.11  [0.00, 1.01], 0.0506 ^b^ | 0.37  [0.20, 0.67], 0.0013* | 0.41  [0.25, 0.67], <0.0010* | 0.36  [0.20, 0.66], 0.0011* |
| Net: Ut + P3L ^a^ |  | 1.45  [0.87, 2.45], 0.1597 |  | 0.93  [0.57, 1.51], 0.7634 | 1.30  [0.77, 2.23], 0.3265 | 0.94  [0.58, 1.54], 0.8117 |
| Net: Ut + UtT ^a^ | 0.00  [0.00, 0.00], <0.0010* | 0.02  [0.01, 0.04], <0.0010* | 0.00  [0.00, 0.00], <0.0010* | 1.43  [0.37, 5.26], 0.5938 | - | - |
| Net: Ut + UtL ^a^ | - | 0.02  [0.01, 0.05], <0.0010* | - | 1.25  [0.34, 4.44], 0.7307 | - | - |
| 1h KD: Yes (Ref: No) | - | - | - | 1.16  [1.11, 1.22], <0.0010* | - | 1.16  [1.11, 1.22], <0.0010* |
| Treated surface contact duration | - | - | - | 1.00  [1.00, 1.00], 0.0230* | 1.00  [1.00, 1.00], 0.0577 ^b^ | 1.00  [1.00, 1.00], 0.0445* |
| * = significant at 5% significance level, ^a^ = net reference P3, ^b^ = borderline significance, ref = reference | | | | | | |

**Table S2 1h knockdown and 24h mortality results for all mosquitoes in tests with all BBnet variants** based on the logistic regression model with a logit link function. Where 95% confidence intervals and *p-values* were adjusted by Dunnett’s multiple comparison test, * = significant at 5% significance level, a = net reference P3, b = borderline significance, f = logistic regression based on the firth method/penalised likelihood and ref = reference**.**

| Term | Mean difference [95% Confidence Interval], p-value | | | | | |
| --- | --- | --- | --- | --- | --- | --- |
|  | 1. **Overall netting** | | 1. **l treated surface** | | **C) Treated surface per square cm** | |
|  | **Contact duration** | **Number of contacts** | **Contact duration** | **Number of contacts** | **Contact duration **** | **Number of contacts** |
| (Intercept) | -652.26  [-1552.14, 247.63], 0.1509 | 4847.47  [-664.50, 10359.44], 0.0831 | -281.55  [-1129.41, 566.32], 0.5037 | 6304.83  [3558.85, 9050.81], <0.0010* | - | 0.09 [0.05, 0. 13], <0.0010* |
| Net: P3 + P3T ^a^ | 976.45  [-221.15, 2174.05], 0.1073 | -3978.16  [-10552.70, 2596.37], 0.2284 | 845.11  [27.24, 1662.98], 0.0433* | -2870.22  [-6079.37, 338.94], 0.0778 | 0.01  [-0.03, 0.05], 0.9666 | -0.05  [-0.10, 0.00], 0.0470* |
| Net: P3 + P3L ^a^ | 1888.65  [750.43, 3026.86], 0.0017* | -933.98  [-6411.52, 4543.57], 0.7281 | 1794.62  [1028.98, 2560.26], <0.0010* | -1858.07  [-4923.00, 1206.87], 0.2259 | 0.02  [-0.02, 0.06], 0.6438 | -0.04  [-0.09, 0.00], 0.0695 |
| Net: P3 + UtT ^a^ | 908.71  [ -293.69, 2111.11], 0.1346 | -3043.99  [-9759.12, 3671.13], 0.3651 | 481.38  [-364.03, 1326.80], 0.2547 | -3545.48  [-6768.82, -322.14], 0.0321* | 0.00  [-0.04, 0.04], 0.9995 | -0.05  [-0.10, 0.00], 0.0417* |
| Net: P3 + UtL ^a^ | 2646.89  [1504.83, 3788.95], <0.0010* | -2225.06  [ -8203.91, 3753.80], 0.4523 | 1691.48  [885.42, 2497.54], <0.0010* | -4504.17  [-7667.04, -1341.31], 0.0067* | 0.02  [-0.02, 0.06], 0.5023 | -0.07  [-0.12, -0.02], 0.0068* |
| Net: Ut + P3T ^a^ | 1106.41  [-33.58, 2246.40], 0.0568 ^b^ | -2786.16  [-8979.44, 3407.13], 0.3683 | 728.46  [-257.68, 1714.60], 0.1422 | -7722.39  [-10783.45, -4661.34], <0.0010* | 0.08  [0.01, 0.14], 0.0119* | -0.09  [-0.13, -0.04], <0.0010* |
| Net: Ut + P3L ^a^ | 3023.08  [1889.50, 4156.66], <0.0010* | 949.42  [-5033.63, 6932.48], 0.7488 | 1465.99  [533.44, 2398.54], 0.0031* | -6959.89  [-10024.82, -3894.95], <0.0010* | 0.06  [0.02, 0.11], 0.0012* | -0.04  [-0.09, 0.01], 0.0986 |
| Net: Ut + UtT ^a^ | 3021.72  [1778.65, 4264.80], <0.0010* | 10282.26  [4247.78, 16316.74], 0.0015* | - | - | - | - |
| Net: Ut + UtL ^a^ | 981.42  [-489.39, 2452.23], 0.1852 | 20357.59  [13549.12, 27166.05], <0.0010* | - | - | - | - |
| Number of contacts | 0.18  [0.13, 0.23], <0.0010* | - | 0.13  [0.05, 0.22], 0.0024* | - | - | - |
| Head: Right (ref: Left) | - | -2811.02  [-5729.85, 107.81], 0.0585^b^ | - | - | - | - |
| Max no.of mosquitoes | - | 1247.64  [354.39, 2140.88], 0.0076* | - | 551.00  [17.01, 1084.99], 0.0435* | - | 0.01  [0.00, 0.02], 0.0212* |
| * = significant at 5% significance level, ^a^ = net reference P3, ^b^ = borderline significance, ref = reference, ** = at the average net number of contacts per square cm i.e. 0.07 (net*number of contacts interaction significant: *F-value* = 4.17, df = 6, p = 0.0045). | | | | | | |

**Table S3 Number and duration of net contacts results by Tiassale mosquitoes in tests with all BBnet variants based on a linear mixed effects model.** Where 95% confidence intervals and *p-values* were adjusted by Dunnett’s multiple comparison test, * = significant at 5% significance level, a = net reference P3, b = borderline significance, ref = reference **and** ** = at the average net number of contacts per square cm i*.e.* 0.07 (net*number of contacts interaction significant: *F-value* = 4.17, df = 6, *p* = 0.0045).at the Permanet 3.

| **Group** | **Net** | **Duration of contact** | | | **Number of contacts** | | |
| --- | --- | --- | --- | --- | --- | --- | --- |
|  |  | **Mean** | **95% Lower CI** | **95% Upper CI** | **Mean** | **95% Lower CI** | **95% Upper CI** |
| Total BBNet area | P3 | 792.16 | 560.59 | 1023.73 | 8049.67 | 3315.70 | 12783.64 |
|  | P3 + P3T | 1256.88 | 295.00 | 2218.76 | 6007.93 | 3387.52 | 8628.35 |
|  | P3 + P3L | 2314.44 | 1125.61 | 3503.27 | 5197.81 | 1105.59 | 9290.04 |
|  | P3 + UtT | 1064.26 | 532.67 | 1595.85 | 5299.50 | 2697.29 | 7901.72 |
|  | P3 + UtL | 2945.56 | 1728.33 | 4162.80 | 4501.89 | 342.40 | 8661.39 |
|  | Ut + P3T | 1468.92 | 642.60 | 2295.24 | 8358.17 | 4216.25 | 12500.08 |
|  | Ut + P3L | 3870.60 | 1353.20 | 6387.99 | 5655.24 | 1997.95 | 9312.52 |
|  | Ut + UtT | 5615.55 | 3470.36 | 7760.74 | 25087.95 | 8086.86 | 42089.04 |
|  | Ut + UtL | 4830.89 | 2234.21 | 7427.57 | 18090.33 | 9294.10 | 26886.57 |
| Total treated area | P3 | 792.16 | 560.59 | 1023.73 | 8049.67 | 3315.70 | 12783.64 |
|  | P3 + P3T | 1256.88 | 295.00 | 2218.76 | 5197.81 | 1105.59 | 9290.04 |
|  | P3 + P3L | 2314.44 | 1125.61 | 3503.27 | 6007.93 | 3387.52 | 8628.35 |
|  | P3 + UtT | 758.98 | 435.84 | 1082.12 | 4191.96 | 228.73 | 8155.18 |
|  | P3 + UtL | 1993.09 | 1203.07 | 2783.12 | 4371.99 | 1921.18 | 6822.80 |
|  | Ut + P3T | 478.32 | 159.44 | 797.20 | 235.44 | 66.24 | 404.65 |
|  | Ut + P3L | 1354.30 | 340.31 | 2368.29 | 1273.45 | 609.11 | 1937.78 |
|  | Ut + UtT | NA | NA | NA | NA | NA | NA |
|  | Ut + UtL | NA | NA | NA | NA | NA | NA |
| Treated area/cm^2^ | P3 | 0.01 | 0.01 | 0.02 | 0.12 | 0.05 | 0.19 |
|  | P3 + P3T | 0.02 | 0.00 | 0.03 | 0.07 | 0.01 | 0.13 |
|  | P3 + P3L | 0.03 | 0.01 | 0.04 | 0.07 | 0.04 | 0.10 |
|  | P3 + UtT | 0.01 | 0.01 | 0.02 | 0.06 | 0.00 | 0.12 |
|  | P3 + UtL | 0.03 | 0.02 | 0.04 | 0.07 | 0.03 | 0.10 |
|  | Ut + P3T | 0.07 | 0.02 | 0.11 | 0.03 | 0.01 | 0.06 |
|  | Ut + P3L | 0.09 | 0.02 | 0.16 | 0.08 | 0.04 | 0.13 |
|  | Ut + UtT | NA | NA | NA | NA | NA | NA |
|  | Ut + UtL | NA | NA | NA | NA | NA | NA |

**Table S4** Descriptive statistics for the number of contacts and contact duration by net variant at different net surfaces (Tiassale strain). Where CI = confidence interval.
